# Digital-Assisted Self-interview of HIV or Sexually Transmitted Infection Risk Behaviors in Transmasculine Adults: Development and Field Testing of the Transmasculine Sexual Health Assessment

**DOI:** 10.2196/40503

**Published:** 2023-03-17

**Authors:** Sari L Reisner, David R Pletta, Dana J Pardee, Madeline B Deutsch, Sarah M Peitzmeier, Jaclyn MW Hughto, Meg Quint, Jennifer Potter

**Affiliations:** 1 Brigham and Women's Hospital Boston, MA United States; 2 Harvard Medical School Boston, MA United States; 3 Harvard T.H. Chan School of Public Health Boston, MA United States; 4 Fenway Health Boston, MA United States; 5 University of California San Francisco San Francisco, CA United States; 6 University of Michigan Ann Arbor, MI United States; 7 Brown University School of Public Health Providence, RI United States; 8 Beth Israel Deaconess Medical Center Boston, MA United States

**Keywords:** transgender, sexual health, HIV, sexually transmitted infection, STI, epidemiology, mobile phone

## Abstract

**Background:**

The sexual health of transmasculine (TM) people—those who identify as male, men, or nonbinary and were assigned a female sex at birth—is understudied. One barrier to conducting HIV- and sexually transmitted infection (STI)–related research with this population is how to best capture sexual risk data in an acceptable, gender-affirming, and accurate manner.

**Objective:**

This study aimed to report on the community-based process of developing, piloting, and refining a digitally deployed measure to assess self-reported sexual behaviors associated with HIV and STI transmission for research with TM adults.

**Methods:**

A multicomponent process was used to develop a digital-assisted self-interview to assess HIV and STI risk in TM people: gathering input from a Community Task Force; working with an interdisciplinary team of content experts in transgender medicine, epidemiology, and infectious diseases; conducting web-based focus groups; and iteratively refining the measure. We field-tested the measure with 141 TM people in the greater Boston, Massachusetts area to assess HIV and STI risk. Descriptive statistics characterized the distribution of sexual behaviors and HIV and STI transmission risk by the gender identity of sexual partners.

**Results:**

The Transmasculine Sexual Health Assessment (TM-SHA) measures the broad range of potential sexual behaviors TM people may engage in, including those which may confer risk for STIs and not just for HIV infection (ie, oral-genital contact); incorporates gender-affirming language (ie, *genital* or *frontal* vs *vaginal*); and asks sexual partnership characteristics (ie, partner gender). Among 141 individual participants (mean age 27, SD 5 years; range 21-29 years; n=21, 14.9% multiracial), 259 sexual partnerships and 15 sexual risk behaviors were reported. Participants engaged in a wide range of sexual behaviors, including fingering or fisting (receiving: n=170, 65.6%; performing: n=173, 66.8%), oral-genital sex (receiving: n=182, 70.3%; performing: n=216, 83.4%), anal-genital sex (receptive: n=31, 11.9%; insertive: n=9, 3.5%), frontal-genital sex (receptive: n=105, 40.5%; insertive: n=46, 17.8%), and sharing toys or prosthetics during insertive sex (n=62, 23.9%). Overall barrier use for each sexual behavior ranged from 10.9% (20/182) to 81% (25/31). Frontal receptive sex with genitals and no protective barrier was the highest (21/42, 50%) with cisgender male partners. In total, 14.9% (21/141) of participants reported a lifetime diagnosis of STI. The sexual history tool was highly acceptable to TM participants.

**Conclusions:**

The TM-SHA is one of the first digital sexual health risk measures developed specifically with and exclusively for TM people. TM-SHA successfully integrates gender-affirming language and branching logic to capture a wide array of sexual behaviors. The measure elicits sexual behavior information needed to assess HIV and STI transmission risk behaviors. A strength of the tool is that detailed partner-by-partner data can be used to model partnership-level characteristics, not just individual-level participant data, to inform HIV and STI interventions.

## Introduction

### Background

Transmasculine (TM) people—those who identify as men, male, transgender men, or a nonbinary gender identity and were assigned female sex at birth [1]—are at risk of HIV and other sexually transmitted infections (STIs), such as gonorrhea, chlamydia, and human papillomavirus (HPV). Historically, research has largely focused on the risk of HIV and STIs in transfeminine populations, that is, those who identify as women, female, transgender women, or a nonbinary gender identity and were assigned male sex at birth [2,3]. Studies on HIV and STI burden in TM people remain scarce [2,4]. Moreover, there is a lack of consistency in the reported rates of STIs in transgender men, with 1 review finding STI ranges of 0% to 4.2% for syphilis, 0% to 10.5% for gonorrhea, and 1.2% to 11.1% for chlamydia [2]. It is important to capture the diverse sexual practices of TM people that may be relevant not only for HIV but also for STIs such as genital or anal HPV, gonorrhea, and chlamydia, as well as for counseling on family planning and contraception needs. Failure to collect complete detail regarding sexual behaviors and the timing and recency of those behaviors may result in misclassification of sexual risk exposures, especially given the heterogeneity of sexual behaviors, gender identities, and the bodies of TM people and their sexual partners.

TM people hold a wide range of sexual identities and engage in a variety of sexual behaviors based on their own and their partner’s anatomy. Analyzing data from the US National Transgender Discrimination Survey, a sample of 2578 TM respondents identified their sexuality as gay (19%), bisexual (13%), and queer (51%) [5], supporting the need for measures that are able to capture the diversity of sexual partners and behaviors. Statistics show that TM people vary in terms of access to, and desire for, genital surgery. In the 2015 US. Transgender Survey, only 5% of TM respondents had a metoidioplasty (detachment of clitoral ligaments to lengthen an enlarged clitoris to create a neophallus) or phalloplasty (creation of a neophallus using extragenital tissue, typically from the forearm or thigh), but >25% and 19% indicated that they “someday” want a metoidioplasty and phalloplasty, respectively [6,7]. Regardless of anatomy, TM people may or may not engage in sexual behaviors that use their own or their partner’s genitals. The wide range of anatomies and sexual identities confers varying sexual behaviors among TM people, which may include higher risk behaviors such as penetrative receptive sex with cisgender gay men that places TM people at risk for the acquisition of HIV and other STIs. Moreover, TM people who experience barriers to surgical access or who do not desire gender-affirming surgery with cervix removal are at risk of cervical cancer caused by high-risk HPV, which can be transmitted or acquired through oral sex, anal sex, and genital-genital contact. Although estimates of HPV are comparable between cisgender women and TM people [8-12], TM people are less likely to receive cervical cancer screenings according to recommended guidelines, underscoring the need for a sexual health measure that allows providers to better understand whether TM patients are at risk of HPV exposure [13]. Other STIs, including gonorrhea, chlamydia, and herpes simplex virus, also warrant attention.

Previous HIV or STI risk surveillance measures, such as the AIDS Risk Behavior Assessment and Risk Assessment Battery, are commonly used and adapted to assess HIV risk [14,15]. However, these measures have limitations. First, these measures use gendered, cisnormative language (eg, assumptions that those who have penises are men and use he or him pronouns), which can be harmful to TM people who are frequently misgendered and systematically misrepresented in data collection [16]. Lack of acceptability of measures in TM populations may lead to nonresponse bias and unwillingness to participate in research or share their sexual history honestly with providers. Furthermore, cisnormative language generates validity concerns when TM people are not able to answer questions accurately according to their or their partners’ anatomies. Second, the measures often do not consider sexual practices that may be more common among TM people, such as the use of prosthetics or toys during sex. These sexual practices are common in TM communities and carry the risk of HPV, gonorrhea, chlamydia, and herpes simplex virus transmission or acquisition but are not assessed by traditional measures. Transgender and gender-diverse activists continue to call for inclusive HIV or STI research measures, notably stating, “A lack of research does not mean we are not at risk” [17,18]. Traditional measures may, therefore, underestimate HIV or STI risk in this population by failing to assess common sexual practices. Third, many measures fail to distinguish between insertive and receptive sexual practices, which have different implications for HIV or STI risk and the probability of transmission or acquisition [19]. To our knowledge, only 1 inclusive sexual health and reproductive health inventory has been published for transgender and nonbinary people, which captures sexual behavior, gender affirmation, pregnancy, abortion, and contraception and is of great use for research but may be too lengthy for providers in clinics to accurately and quickly assess HIV or STI risk in only TM people [20]. A study by Bauer et al [21] exploring the sexual health of TM people who have sex with men indicated that they developed and used measures that include a wide range of sexual behaviors, but they did not provide structure or enough details to accurately recreate those measures.

There is limited knowledge and lack of consensus about how best to capture sexual risk data in the TM population, both in terms of accurately characterizing sexual behaviors that confer specific risks for HIV and STI transmission and ensuring that the measure is gender affirming and acceptable for TM respondents. Digital-assisted self-interviewing uses computer- or technology-deployed methods (eg, laptops, electronic tablets, and smartphones) to collect survey data, wherein the respondent completes the assessment via self-reporting without an interviewer administering it. The capacity for programmed branch logic and the highly sensitive nature of sexual risk behavior questions makes this a method well suited for confidential data collection of self-reported HIV- and STI-related risks for TM adults [22,23].

### Objectives

This study aimed to develop and test a robust and inclusive sexual health and risk assessment designed *with* and specifically *for* TM people. The aims were twofold: (1) to report on the community-based process of developing and refining a digital-assisted self-interview measure designed to assess self-reported sexual behaviors associated with HIV and STI transmission for research in TM adults, known as the Transmasculine Sexual Health Assessment (TM-SHA), and (2) to field and digitally implement the TM-SHA and characterize the sexual histories, sexual partnership, and sexual practices in a sample of TM adults.

## Methods

### Overview

This research was undertaken from March 2015 to September 2016 as part of a biobehavioral study to evaluate screening methods for the detection of high-risk HPV in TM adults (ClinicalTrials.gov NCT02401867) [24]. The main outcomes and broader study details have been reported elsewhere [8]. This secondary analysis focuses on the development, implementation, and refinement of the HIV and STI sexual risk measure, including reporting on HIV and STI transmission risk by the gender of sexual partners.

### Ethics Approval

All study activities were approved by the Fenway Health Institutional Review Board (FWA00000145).

### Consent to Participate

Informed consent procedures ensured that the people understood what was involved in participating in the study, what the risks and benefits were of participation, and voluntarily agreed to participate. The original informed consent also applied to this secondary analysis, for which all study data were anonymized and deidentified. Participants were compensated US $100 for study participation in the form of a prepaid American Express gift card.

### Community-Scientific Partnership

A multicomponent community-based process was used to develop and refine a questionnaire to assess HIV and STI risk in TM people using digital-assisted self-interviewing technology. The process used community-based participatory research principles to work *with* not *on* TM communities and to cocreate and refine the sexual behavior assessment. The development process was guided by a 15-member research team that included multiple TM adults who worked closely with a 5-member Community Task Force of TM people and a 5-member multidisciplinary Scientific Advisory Board that comprised content experts in transgender medicine, clinical epidemiology, and infectious diseases research. This was a dynamic process involving community and scientific engagement. At the core of the enterprise was the following tenet: to prioritize being gender affirming and respectful, while collecting accurate data in a patient-centered manner to understand and address sexual health needs of TM people.

### Development, Modification, and Refinement of the Measure

The TM-SHA was developed to assess sexual behaviors, partnerships, and protective barrier use that confer transmission risk of HIV and other STIs. The primary purpose was to measure the broad range of sexual behaviors engaged in by TM adults, including those that may confer risk for HIV, HPV, and other STIs, in a gender-affirming, sensitive, and accurate manner.

The basic TM-SHA assessment structure was modeled on an HIV risk assessment originally adapted from the AIDS Risk Behavior Assessment [14] by the LifeSkills trial, a multisite study testing the efficacy of an HIV behavioral intervention to reduce HIV transmission risk for young transgender women aged 16-29 years [20]. The measure was subsequently adapted with young adult transgender men who have sex with men aged 18-29 years in LifeSkills for Men [25]. These HIV risk measures specifically focused on sexual and other behaviors that confer risk for HIV infection, such as genital and anal intercourse and needle sharing in the context of injection drug use or hormone therapy. This study expanded on this to include a broader range of behaviors.

The development of the TM-SHA involved active collaboration between the research team, Task Force, and Scientific Advisory Board to ensure the creation of a gender-affirming assessment that reflected the sexual lives and experiences of TM adults. Among these groups, we discussed the topics of language and terminology, usability, question clarity and specificity, response options, and acceptability. A preliminary version of the TM-SHA was then pilot-tested with members of the Task Force and the research team, who provided feedback regarding the instrument’s functionality and design. After addressing feedback from the pilot test, we field-tested the TM-SHA in a sample of 141 TM adults, all of whom had cervixes and reported at least 1 sexual partner in the past 12 months (see *Field Test of the Assessment* section).

When reviewing the preliminary version of the measure, the following ideas emerged about the components of the ideal assessment: asking about an array of sexual behaviors, including those that are not typically associated with HIV risk; adding an introduction about the complexity of terminology; asking participants’ surgical status and programming skip patterns to assess sexual risk accordingly; asking participants what language they use to describe their body parts and then having that populate subsequent questions about sexual behaviors and risks; and assessing sexual partner and partnership characteristics.

On the basis of these ideas, the following changes and refinements to the instrument were made: adding questions to assess the broad range of potential sexual behaviors TM people may engage in, including those which may confer risk for HPV and other STIs, not only for HIV infection; incorporating gender-affirming language to make questions and response options less gendered and binary sex specific (ie, using more generic terms like *genital* vs *vaginal*); and asking sexual partner and partnership characteristics to understand interpersonal context.

### TM-SHA Measure

The TM-SHA measure is summarized in Table 1, and Table 2 presents the grid of sexual behaviors and frequency of barrier use (the full TM-SHA measurement and survey logic guidelines can be obtained by contacting the corresponding author). The TM-SHA assessed individual- and partnership-level risks. At the individual level, participants were asked to provide information about their sexual orientation and the total number of sexual partners in the past 36 months.

Participants were then asked to provide demographic and sexual risk–related data for up to 3 sexual partnerships from the past 12 months. These partnership-level data included the gender identity of the sexual partner, configuration of the sexual partnership, behaviors engaged in during sex, frequency of protective barrier use by sexual behavior, and whether the sexual partner had ever been diagnosed with an STI.

Sexual behavior data collected through the TM-SHA consisted of the following 15 sexual behaviors: (1) performing frontal (ie, vaginal) and/or anal penetration with a finger or fist, (2) receiving frontal and/or anal penetration with a finger or fist, (3) performing oral-genital sex, (4) receiving oral-genital sex, (5) performing oral-anal sex, (6) receiving oral-anal sex, (7) frontal insertive sex with genitals, (8) frontal receptive sex with genitals, (9) frontal insertive sex with a toy or prosthetic, (10) frontal receptive sex with a toy or prosthetic, (11) anal insertive sex with genitals, (12) anal receptive sex with genitals, (13) anal insertive sex with a toy or prosthetic, (14) anal receptive sex with a toy or prosthetic, and (15) insertive sex in which a toy or prosthetic was shared.

The use of a protective barrier can be operationalized from the measure as using a condom, internal condom, dental dam, or gloves during sexual activity. The frequency of protective barrier use can be coded as either engaged in sexual behavior with no use of a protective barrier or engaged in sexual behavior and used a protective barrier on at least 1 occasion.

**Table 1:** Overview of the Transmasculine Sexual Health Assessment.

Section, section #, and item				Type
**Introduction**				
	1	This part of the survey will ask you about sex. Sex is a personal issue that can sometimes be sensitive or difficult to talk about, especially for those who are transgender or gender nonconforming because bodies do not always reflect identity. We will be asking you about different forms of sexual activity including the following: penetrative sex with fingers (“fingering”) or fists (“fisting”), oral-genital sex (putting your mouth on someone’s genitals or someone putting their mouth on your genitals), oral-anal sex (“rimming”), receptive and insertive frontal sex, receptive and insertive anal sex, and use of prosthetics/toys. By “receptive sex” we mean when a person inserts their genitals or a toy into your frontal opening or anus. By “insertive sex” we mean when your genitals are inserted into a frontal opening or anus without the assistance of a prosthetic/dildo/toy/etc. We will also be asking you about your use of barriers during sexual activity. Barriers differ depending on the sexual activity. Barrier methods include condoms, internal condoms, dental dams, and gloves. Except where indicated, all questions about sex refer to consensual sex or sex you experienced because you wanted to participate and not because you were forced, coerced, or otherwise made to have sex. Remember your answers to these questions will be kept completely private. Please try your best to answer each question.	Prompt	
**Demographics**				
	1	Please answer the following questions about your sexual identity, attractions, and recent sexual activity.	Prompt	
	2	Which of the following best describes your sexual identity or orientation today? Choose one... (1) gay/homosexual/same-gender attraction; (2) straight/heterosexual; (3) bisexual; (4) queer; (5) pansexual; (6) questioning; (7) asexual; (8) unsure; (9) I do not label my sexual orientation; (97) Other, please specify; and (99) I prefer not to answer.	Multiple choice	
**Sexual contact (last 36 months)**				
	1	Please think about the people you have had sexual contact with in the last 36 months (3 years).	Prompt	
	2	How many individuals have you had any form of sexual contact with in the last 36 months (3 years)? Sexual contact includes penetrative sex using fingers or fists, oral-genital sex, oral-anal sex, receptive and/or insertive frontal sex, receptive and/or insertive anal sex, and use of prosthetics/toys. If you would prefer not to answer, please enter “99,999.”	Open response	
	3	Please specify the gender(s) of the partner(s) you have had sexual contact with in the last 36 months (3 years) and the number of partner(s) of each gender. The total must be equal to the number you specified in the previous question. If you did not have sexual contact with a person of a specified gender, please enter 0. If you would prefer not to answer, please enter “99,999”; in each field that you do not wish to answer ... (1) cisgender/nontransgender man, (2) cisgender/nontransgender woman, (3) transgender man (FTM^a^), (4) transgender woman (MTF^b^), (5) male-assigned gender nonconforming/nonbinary person, and (6) female-assigned gender nonconforming/nonbinary person.	Multiple choice	
	4	Of the partners that you had sexual contact with in the last 36 months (3 years), how many did you have unprotected frontal and/or anal sex with (ie, without a condom or other barrier)? This includes penetrative sex using fingers or fists, oral-genital sex, oral-anal sex, receptive and/or insertive frontal sex, receptive and/or insertive anal sex, and use of prosthetics/toys. If you would prefer not to answer, please enter “99,999.”	Open response	
**Sexual contact (last 12 months)**				
	1	Please think about the people you have had sexual contact with in the last 12 months (1 year).	Prompt	
	2	How many individuals have you had any form of sexual contact with in the last 12 months (1 year)? Sexual contact includes penetrative sex using fingers or fists, oral-genital sex, oral-anal sex, receptive and/or insertive frontal sex, receptive and/or insertive anal sex, and use of prosthetics/toys. If you would prefer not to answer, please enter “99,999.”	Open response	
	3	Please specify the gender(s) of the partner(s) you have had sexual contact with in the last 12 months (1 year) and the number of partner(s) of each gender. The total must be equal to the number you specified in the previous question. If you did not have sexual contact with a person of a specified gender, please enter 0. If you would prefer not to answer, please enter “99,999”; in each field that you do not wish to answer... (1) cisgender/nontransgender man, (2) cisgender/nontransgender woman, (3) transgender man (FTM), (4) transgender woman (MTF), (5) male-assigned gender nonconforming/nonbinary person, and (6) female-assigned gender nonconforming/nonbinary person.	Multiple choice	
	4	Of the partners that you had sexual contact with in the last 12 months (1 year), how many did you have unprotected frontal and/or anal sex with (ie, without a condom or other barrier)? This includes penetrative sex using fingers or fists, oral-genital sex, oral-anal sex, receptive and/or insertive frontal sex, receptive and/or insertive anal sex, and use of prosthetics/toys. If you would prefer not to answer, please enter “99,999.”	Open response	
**Identifying number of sexual partners (last 12 months)**				
	1	We will now ask you about your 3 most recent sexual partners in the last 12 months (1 year).	Prompt	
	2	Thinking about the people that you have had sexual contact with in the last 12 months, please enter the initials of your most recent sexual partner. If you do not know the person’s initials or do not feel comfortable providing that information, please create your own way of identifying this partner. ^c^Note: if you have had sex with >1 partner at the same time, please choose only 1 partner for this question, and use the next question(s) to identify the other, concurrent partner(s).	Open response	
	3	Thinking about the people that you have had sexual contact with in the last 12 months, did you have another partner in addition to (partner 1 initials)? ... (1) yes, (2) no, and (99) I prefer not to answer.	Multiple choice	
	4	(If yes) Please enter the initials of this sexual partner. If you do not know the person’s initials or do not feel comfortable providing that information, please create your own way of identifying this partner.	Open response	
	5	(If sex partners last 12 months >2) Thinking about the people that you have had sexual contact with in the last 12 months, did you have another partner in addition to (partner 1 initials) and (partner 2 initials)? ... (1) yes, (2) no, and (99) I prefer not to answer.	Multiple choice	
	6	(If yes) Please enter the initials of this sexual partner. If you do not know the person’s initials or do not feel comfortable providing that information, please create your own way of identifying this partner.	Open response	
**Sexual partner one**				
	1	Please answer the following questions about your interactions with (partner 1 initials).	Prompt	
	2	What was (partner 1 initials)’s gender? ... (1) cisgender/nontransgender man, (2) cisgender/nontransgender woman, (3) transgender man (FTM), (4) transgender woman (MTF), (5) male-assigned gender nonconforming/nonbinary person; (6) female-assigned gender nonconforming/nonbinary person, and (99) I prefer not to answer.	Multiple choice	
	3	How would you describe your relationship with (partner 1 initials)? If >1 description applies, please select “Other” and describe... (1) married or in a civil partnership; (2) serious relationship (boyfriend/girlfriend/partner), someone you dated for a while and feel very close to; (3) casually dating but not serious; (4) poly (polyamorous); (5) open relationship/nonmonogamous; (6) sleeping with this person (“fuck buddy” or “booty call”) but not dating; (7) dom/sub (dominant/submissive); (8) fluid bonded; (9) one night stand; (10) stranger or anonymous person; (11) exchange partner/sex work client; (97) other, please specify; and (99) I prefer not to answer.	Multiple choice	
	4	For the following questions, please identify whether or not you engaged in any of the following sexual activities in the last 12 months with this partner (partner 1 initials). If you respond “Yes,” please specify how often a barrier was used. If you respond “No,” please select “I did not engage in this activity” under barrier use. ^c^Note: as a reminder, by “barrier” we mean condoms, internal condoms, dental dams, and gloves.	Prompt	
	5	Sexual behaviors and frequency of protective barrier use grid^d^	Multiple choice	
	6	STI^e^ history^d^: Has this partner ever been diagnosed (by a physician, nurse, or other medical provider) with any of the following STIs?: (1) HIV, (2) HPV^f^, or (3) HSV^g^ type 1 or 2 (response options: yes, no, I do not know, and I prefer not to answer).	Multiple choice	

^a^FTM: female-to-male.

^b^MTF: male-to-female.

^c^The text following “Note” was presented as clarification for participants completing the survey.

^d^See Table 2 for the grid of sexual behaviors and protective barrier use.

^e^STI: sexually transmitted infection.

^f^HPV: human papillomavirus.

^g^HSV: herpes simplex virus.

**Table 2:** Transmasculine Sexual Health Assessment: grid of sexual behaviors and frequency of protective barrier use.

	Activity performed with (initials of sexual partner)^a^ in the last 12 months?				While engaged in this activity, how often was a barrier used?						
	Yes	No	I prefer not to answer	I did not engage in this activity		Never	Less than half the time	About half the time	More than half the time	Always	I prefer not to answer
Frontal and/or anal penetration with a finger (“fingering”) or fist (“fisting”) - performed											
Frontal and/or anal penetration with a finger (“fingering”) or fist (“fisting”) -received											
Oral-genital – performed											
Oral-genital – received											
Oral-anal (“rimming”) - performed											
Oral-anal (“rimming”) - received											
Frontal receptive - with genitals											
Frontal receptive - with toy or prosthetic											
Frontal insertive - with genitals											
Frontal insertive - with toy or prosthetic											
Anal receptive - with genitals											
Anal receptive - with toy or prosthetic											
Anal insertive - with genitals											
Anal insertive - with toy or prosthetic											
Insertive sex toys or prosthetics shared											

^a^Participants were asked to list the initials for each of their most 3 recent sexual partners in the last 12 months (Table 2). The initials for each sexual partners were prepopulated into the assessment grid for sexual behaviors.

### Field Test of the Measure

The TM-SHA was subsequently deployed via a digital tablet in a sample of 141 TM people aged 21 to 50 years in the greater Boston, Massachusetts, area. Participants were recruited via convenience sampling methods (eg, flyers and word-of-mouth referral) and through medical providers. Study visits were held at a community health center that specialized in gender-affirming transgender care in Boston, Massachusetts [26], and lasted for approximately 3 hours. During the study visit, the participants completed a 45-minute cross-sectional survey in which the TM-SHA was embedded. The details of the study protocol have been described elsewhere [24]. After the completion of the TM-SHA measure, the participants were asked to provide optional qualitative, open-ended feedback about their experience with the assessment. The TM-SHA required 20 minutes to complete.

### Data Analysis

Descriptive analyses characterized the distribution of TM adults’ sexual behaviors and HIV and STI transmission risk by the gender identity of sexual partners. The gender identity of sexual partners was coded into the following 4 groups: TM (combining transgender man and assigned female gender nonconforming or nonbinary person), transfeminine (combining transgender woman and assigned male gender nonconforming or nonbinary person), cisgender man, and cisgender woman. Descriptive statistics for the sample were calculated using TM adults as individual participants (N=141). This participant-level data set was then transposed from *short* to *long* formatting to reorient the sexual partnership data (n=259 partnerships). As participants provided data for up to 3 sexual partnerships within the past 12 months, the long-formatted data set allowed us to descriptively analyze protective barrier use within the sexual partnership data. Protective barrier use was operationalized as using a condom, internal condom, dental dam, or gloves during sexual activity versus not using the aforementioned protective barriers. Statistical analyses were conducted using SAS (version 9.4; SAS Institute). Themes from the open-ended feedback from the participants on the TM-SHA were summarized qualitatively in Excel (Microsoft Corporation).

### Data Exclusion

In total, 9 participants were excluded from the data analysis because they did not report any partnership-level data.

## Results

### Sample Characteristics

The characteristics of participants (N=141) are presented in Table 3. The majority were aged between 21 and 29 years (102/141, 72.3%), with a mean age of 27 years. Approximately 75.2% (106/141) and 14.9% (21/141) of the participants were White and multiracial, respectively. Among all the participants, approximately 14.9% (21/141) reported a personal history of STI diagnosis.

**Table 3:** Demographic characteristics of transmasculine adults in field-testing sample (N=141).^a^

Characteristics			Values^b^
**Age (years), n (%)**			
	21-24	45 (31.9)	
	25-29	57 (40.4)	
	30-34	25 (17.7)	
	≥35	14 (9.9)	
Age (years), mean (SD)			27.4 (5.7)
**Race, n (%)**			
	White	106 (75.2)	
	Black	4 (2.8)	
	Asian	8 (5.7)	
	Native Hawaiian or other Pacific Islander	1 (0.1)	
	Multiracial	21 (14.9)	
	Missing	1 (0.71)	
**Gender identity, n (%)**			
	Man or male	38 (26.9)	
	Transgender man (Female-to-Male)	71 (50.4)	
	Genderqueer or nonbinary	27 (19.2)	
	Another gender^a^	5 (3.5)	
**Sexual orientation, n (%)**			
	Gay, homosexual, or same-gender attraction	14 (9.9)	
	Straight or heterosexual	16 (11.4)	
	Bisexual	18 (12.8)	
	Queer	65 (46.1)	
	Pansexual	13 (9.2)	
	Asexual	3 (2.2)	
	Questioning or unsure	3 (2.1)	
	I do not label my sexual orientation	7 (5.0)	
	Missing	2 (1.4)	
**Lifetime STI^c^ diagnosis^d^, n (%)**			
	No	120 (85.1)	
	Yes	21 (14.9)	
Number of sexual partners within the past 12 months, median (IQR)			2 (3)

^a^Includes agender, bigender, and write-in gender identities distinguishable from the categories provided.

^b^Percentages may not sum to 100% owing to rounding.

^c^STI: sexually transmitted infection.

^d^Includes any lifetime diagnosis of HIV, chlamydia, trichomoniasis, syphilis, gonorrhea, genital herpes, hepatitis B, hepatitis C, or another STI.

### Sexual Partnership Characteristics

In Table 4, descriptive statistics of sexual partnerships (n=259) and practices (15 sexual risk behaviors) are shown, along with the frequency of protective barrier use by sexual behaviors. The participants engaged in a wide range of sexual behaviors, including fingering or fisting (receiving: 170/259, 65.6% and performing: 173/259, 66.8%), oral-genital sex (receiving: 182/259, 70.3% and performing: 216/259, 83.4%), anal-genital sex (receptive: 31/259, 11.9% and insertive: 9/259, 3.5%), frontal-genital sex (receptive: 105/259, 40.5% and insertive: 46/259, 17.8%), and sharing toys or prosthetics during insertive sex (62/259, 23.9%). The overall barrier use for each sexual behavior varied greatly, ranging from 10.9% (20/182) to 81% (25/31).

The prevalence of protective barrier use was heterogeneous across different partnerships and sexual behaviors. For example, frontal receptive sex with genitals with no protective barrier ranged from 2% (1/42; with a transfeminine person) to 50% (21/42; with a cisgender man). In our field test of TM-SHA, we found that 40% (42/105) of participants engaged in frontal receptive sex with genitals without the use of a protective barrier. Of those, more than half (22/42, 52%) engaged in this behavior with a transfeminine person or a cisgender man, which may confer risk for HPV and other STIs. One-third (14/42, 33%) of TM participants reported engaging in frontal receptive sex with genitals and no protective barrier with a cisgender woman partner, which is notable given that HPV is also spread by people without a penis. Notably, TM people engaged in insertive frontal and insertive anal sex, in which an enlarged clitoris (often achieved through hormone use) is inserted into a partner’s frontal or anal cavity.

**Table 4:** Characterizing the sexual partners, sexual behaviors, and protective barrier use of transmasculine adults (n=259 partnerships) by gender identity of sexual partners and protective barrier use across varied sexual behaviors.

Sexual behavior	No protective barrier used, n (%)						Protective barrier used, n (%)							Total^a^, n (%)
	Values^b^	Gender of sexual partners					Values^b^		Gender of sexual partners					
		Transgender masculine^c,d^	Transgender feminine^d,e^	Cisgender man^d^	Cisgender woman^d^			Transgender masculine^c,d^		Transgender feminine^d,e^	Cisgender man^d^	Cisgender woman^d^		
Performing frontal and/or anal penetration with a finger or fist	132 (76.3)	21 (16)	6 (5)	20 (15)	85 (64)	41 (24)		16 (39)		4 (10)	3 (7)	18 (44)	173 (66.8)	
Receiving frontal and/or anal penetration with a finger or fist	134 (78.8)	19 (14)	11 (8)	51 (38)	53 (40)	36 (21)		14 (39)		2 (6)	4 (11)	16 (44)	170 (65.6)	
Performing oral-genital sex	184 (85.2)	30 (16)	11 (6)	60 (33)	83 (45)	32 (15)		1 (3)		8 (250)	13 (41)	10 (31)	216 (83.4)	
Receiving oral-genital sex	162 (89)	28 (17)	11 (7)	50 (31)	73 (45)	20 (11)		4 (20)		4 (20)	1 (5)	11 (55)	182 (70.3)	
Performing oral-anal sex	39 (80)	4 (10)	3 (8)	15 (38)	17 (44)	10 (20)		1 (10)		4 (40)	0	5 (50)	39 (15)	
Receiving oral-anal sex	34 (77)	8 (24)	1 (3)	15 (44)	10 (29)	10 (23)		3 (30)		2 (20)	0	5 (50)	44 (17)	
Frontal insertive sex with genitals	29 (63)	6 (21)	0	0	23 (79)	17 (37)		2 (12)		3 (18)	8 (47)	4 (24)	46 (18)	
Frontal receptive sex with genitals	42 (40)	6 (14)	1 (2)	21 (50)	14 (33)	63 (60)		2 (3)		14 (22)	45 (71)	2 (3)	105 (40.5)	
Frontal insertive sex with toy or prosthetic	44 (39)	8 (18)	1 (2)	3 (7)	32 (73)	69 (61)		23 (33)		3 (4)	1 (1)	42 (61)	113 (43.6)	
Frontal receptive sex with toy or prosthetic	41 (47)	10 (24)	3 (7)	10 (24)	18 (44)	46 (53)		13 (28)		5 (11)	4 (9)	24 (52)	87 (34)	
Anal insertive sex with genitals	2 (22)	0	0	1 (50)	1 (50)	7 (78)		2 (29)		1 (14)	2 (29)	2 (29)	9 (3)	
Anal receptive sex with genitals	6 (19)	0	1 (17)	5 (83)	0	25 (81)		0		4 (16)	21 (84)	0	31 (12)	
Anal insertive sex with toy or prosthetic	17 (33)	1 (6)	6 (35)	7 (41)	3 (18)	34 (67)		8 (24)		7 (21)	10 (29)	9 (26)	51 (20)	
Anal receptive sex with toy or prosthetic	11 (31)	3 (27)	2 (18)	4 (36)	2 (18)	25 (69)		8 (32)		3 (12)	3 (12)	11 (44)	36 (14)	
Insertive sex in which toys or prosthetics were shared	21 (34)	3 (14)	2 (10)	2 (10)	14 (67)	41 (66)		14 (34)		3 (7)	4 (10)	20 (49)	62 (24)	

^a^The denominator for percentages in the column is the total number of sexual partnerships (N=259).

^b^The denominator for percentages in the column is the total N for that sexual behavior.

^c^Includes transgender men and gender nonbinary assigned-female-at-birth partners.

^d^The denominator for percentages in the column is the N for barrier use or no barrier use.

^e^Includes transgender women and gender nonbinary assigned-male-at-birth partners.

### Lessons Learned From Participants’ Open-ended Feedback

Through open-ended feedback in the development and field-testing of TM-SHA, the team learned many lessons for measuring TM people’s sexual risk (Textbox 1). The TM-SHA had high levels of acceptability among TM participants. No length-related concerns or participant burden concerns were reported for the measure. Skip logic ensured that participants circumvented any questions that were not relevant to them. Themes that emerged in the open-ended feedback after the completion of the TM-SHA included appreciation expressed by multiple participants for the measure’s gender-affirming language and for the comprehensiveness of the assessment and how it asked about diverse behaviors and partnerships. Two participants noted confusion surrounding the terms “receptive” and “insertive” for describing some of the sexual behaviors and suggested further clarification in the future.

**Lessons learned in measuring sexual risk in transmasculine (TM) adults.**

- Partner and work with TM community members to ensure gender-affirming, cultural competency, and responsiveness of measures.
- Use introductory language at the beginning to acknowledge, validate, and affirm differences in identity and language use for TM people. Specify terms to be used throughout the survey for body parts, sexual acts, etc, for example, the use of *frontal* sex instead of *vaginal* sex.
- Use simple concrete language, which can be challenging with jargon and sexual health terminology. For TM people, carefully select words and phrases to ensure item clarity, similar interpretation of the item across respondents, response options, and choices (eg, check all, mutually exclusive, and open ended).
- Consider the potential emotional impact that terminology and language may evoke and acknowledge sensitivity and constant evolution of language in TM communities.
- Capture the heterogeneity of sexual identities in TM people, diversity of sexual partnership types and behaviors, and interpersonal contexts.
- Validate all sexual practices TM people engage in, regardless of whether they confer high probability of HIV or sexually transmitted infection transmission risk, by asking about high-, low-, and varying-risk activities.
- Do not assume that TM people are limited to certain types of sex based on their gender identity or based on their anatomy (eg, questions should ask about insertive and receptive sex). For example, given the diversity of bodies, it was important to specify whether a sexual act was performed with or without a prosthetic or toy. Gender of one’s sexual partner may not always correspond with their genitals.
- Provide space for open-ended feedback about the assessment and experience of completing the survey. For example, ask participants: Is there anything else you feel to be important that we did not address?

## Discussion

### Principal Findings

The TM-SHA is one of the first digital sexual health risk measures developed specifically with and exclusively for TM people. It successfully integrated gender-affirming language and branching logic that captures the wide array of sexual behaviors and partnerships in TM people. The TM-SHA was highly acceptable to TM adults, with no participant concerns regarding burdensomeness or length of the measure, and it was found to elicit the sexual behavior information needed to assess HIV or STI transmission risk behaviors. For the parent study’s outcome of high-risk HPV, the measure included questions to better assess potential sexual exposures for other STIs besides HIV infection, given that multiple behaviors confer risk for high-risk HPV.

In collaboration with a Task Force and community input, the TM-SHA was adapted with gender-affirming language to measure the sexual behavior and protective barrier use of participants. The use of gender-affirming language may have 2 advantages for measuring the sexual risk behavior of TM people. First, gender-affirming language may improve the validity of the TM-SHA by providing a description of sexual behaviors and genitals in a way that better reflects some of the perspectives of TM people (eg, using *frontal* receptive sex instead of *vaginal* receptive sex) [27]. Second, the gender identity options listed on the TM-SHA reflect those commonly held by TM people and their sexual partners; the instrument served to further acknowledge and validate these gender identities.

The sexual health of TM people remains understudied [2,4], and a barrier to conducting HIV- and STI-related research with this study population is the lack of consensus on how to best capture sexual risk data in a standardized manner that is gender affirming, culturally responsive, and accurate. In the context of widespread stigma facing TM people, there is a need for high-quality measures that are cocreated with communities to maximize acceptability, for example, by avoiding stigmatizing language that may trigger medical and research mistrust and by cueing participants that researchers are knowledgeable and transcompetent in asking sensitive questions. Thus, this study fills an important gap for both researchers and clinicians aiming to capture the wide variety of sexual behaviors of TM people.

The TM-SHA provides a high level of specificity about sexual practices, some of which may confer greater risk for HIV and STIs than others. Additional research is warranted on how to optimize use and interpret the measure. For example, it may be helpful to develop a *scoring* schema to categorize someone for research as *low, moderate, or high* risk based on their reported protection of sexual behaviors or for clinical settings to appropriately counsel a patient on their STI risk or need for Papanicolaou testing. The growing functionality of electronic health records (EHRs) and patient portals offers an opportunity to integrate the TM-SHA and similar tools into clinical care provision. Patients could be asked to complete the TM-SHA before their visit via the patient portal, and the information elicited could be linked to the EHR and integrated into clinical care delivery.

### Limitations and Future Directions

With regard to limitations, the current TM-SHA does not consider HIV prevention methods such as pre-exposure prophylaxis (PrEP) or postexposure prophylaxis to prevent HIV acquisition used by participants or their partners. TM people engage in sexual behaviors conferring eligibility for PrEP but have a low uptake of PrEP [28]. Future iterations of the measure may consider adding questions and skip patterns to assess current PrEP and postexposure prophylaxis use for both TM people and their partners. In addition, the current TM-SHA assumes that the participant had a cervix per eligibility criteria for the parent study. Future versions of the measure may consider adding questions and logic to assess gender-affirming surgeries, which may result in different anatomy and, therefore, sexual risks in participants. According to participant feedback, future versions of the measure would benefit from clarifying insertive and receptive sex. For example, in reporting on frontal receptive sex with genitals with a cisgender woman partner, it will be important to learn how people understand or interpret the terms *receptive* and *with genitals*. Cognitive interviewing methods may assist with this process and strengthen the evidence of validity.

The length of the survey and advanced skipped patterns used in the survey, which may be difficult for beginners to code, are best suited for preprogrammed electronic surveys. This may pose a challenge for those with low literacy rates and for medical providers hoping to use this measure in clinic [22]. An audio, digital-assisted self-interview or interviewer-administered assessment may be used to address the low literacy levels of respondents. Researchers and clinical providers may also adapt and shorten the TM-SHA for their research, clinical practice, and patient needs. For example, if HIV risk is the parameter of interest, the assessment could be shortened to only those sexual behaviors that confer a high HIV risk. Future research is needed to reduce the scope of the tool, ensure its accuracy, and externally validate the measure for clinical robustness. Such research could examine clinical outcomes (eg, laboratory or clinically confirmed STIs) alongside patient-reported outcomes and/or gather and compare patient-reported and provider-documented sexual behavior data for accuracy.

Finally, despite efforts to capture all types of sexual behaviors and partnerships, it is possible that not all behaviors were captured by the measure, as currently designed. Thus, open-ended questions at the end of the measure were added. This allows participants the space to disclose any other relevant information, including the behaviors they may want to report, which may be used in data analysis or to improve the measure for future use. A strength of the TM-SHA is that it is inclusive of polyamorous relationships in that for each partnership they report on, participants are asked to describe the type of partnership (eg, casual or polyamorous). In addition, although participants were not asked directly about multipartner sex (eg, group sex), the assessment asked about sexual behaviors with multiple partners. Asking explicitly about multipartner sex is recommended in the future.

### Conclusions

This study offers a digital measure designed with and specifically for TM people for future use in TM sexual health research and clinical care. The tool garnered high levels of acceptability from TM participants, was feasible to implement in field-testing, and reported no concerns about participant burden or length in completing the assessment. A strength of this measure is that detailed partner-by-partner data can be used to model partnership-level characteristics in addition to individual-level participant data. Additional psychometric evaluation of the measure is necessary, including longitudinal data, to assess the performance of the TM-SHA over time for research purposes. Clinically, the TM-SHA provides a standardized sexual history measure for TM people, which may help clinicians enhance trust with their patients and more effectively identify sexual behaviors that warrant HIV or STI screening in clinical care. Integration of the measure into clinical research and care via patient portals linked to EHR may facilitate the delivery of culturally responsive and gender-affirming sexual health care.

## Abbreviations

EHR: electronic health record
HPV: human papillomavirus
PrEP: pre-exposure prophylaxis
STI: sexually transmitted infection
TM: transmasculine
TM-SHA: Transmasculine Sexual Health Assessment

## Data Availability

The data sets analyzed during this study are available from the corresponding author upon reasonable request.
